# Hemodynamic responses to emotional auditory stimuli in patients with prolonged disorders of consciousness: an fNIRS study

**DOI:** 10.3389/fneur.2025.1631609

**Published:** 2025-09-03

**Authors:** Yuxuan Zhang, Bixuan Duan, Hongxing Cui, Chuanhua Zhu, Binbin Huang, Hongwei Li, Wei Li

**Affiliations:** ^1^School of Special Education and Rehabilitation, Binzhou Medical University, Yantai, China; ^2^Department of Rehabilitation, Binzhou Medical University Hospital, Binzhou, China

**Keywords:** prolonged disorders of consciousness, functional near-infrared spectroscopy, auditory stimulation, emotional processing, prefrontal cortex

## Abstract

**Background:**

In this study, we used functional near-infrared spectroscopy (fNIRS) to detect changes in cerebral blood flow in response to emotional auditory stimuli in patients with prolonged disorders of consciousness (pDoC). We aimed to verify whether hemodynamic responses to emotional auditory stimuli in pDoC patients measured via fNIRS differed significantly depending on the level of consciousness.

**Methods:**

fNIRS was used to assess brain function in 60 subjects, including 20 patients with unresponsive wakefulness syndrome (UWS), 19 patients in a minimally conscious state (MCS), and 21 healthy controls (HC). All the participants were exposed to emotional auditory stimuli, including the subject’s own name (SON), fear stimulus and infant crying stimulus. We identified the mean and slope value as biomarkers reflecting changes in brain function.

**Results:**

For the SON stimulus, mean HbO concentration analysis showed that the UWS group had significantly lower mean HbO concentration changes in the bilateral middle temporal gyrus (MTG), retrosubicular area (RSA), premotor and supplementary motor area (SMA), and specific channels of the right temporopolar area (TPA) and pars triangularis Broca’s area (PTG) compared to the HC group. The MCS group exhibited a significantly lower mean HbO concentration change in the right MTG compared to the HC group and a significantly greater change in the left SMA compared to the UWS group. No significant differences were found in the slope analysis across the three groups. For the fear stimulus, mean oxyhemoglobin (HbO) concentration analysis showed that the MCS group had significantly greater mean HbO concentration changes in the left TPA and dorsolateral prefrontal cortex (DLPFC) compared to the HC group. In the slope analysis, both the UWS and MCS groups exhibited significantly greater values in the left DLPFC compared to the HC group. For the infant crying stimulus, the slope analysis showed that the MCS group had significantly greater values in the frontopolar area (FPA) and left TPA compared to the HC group. However, no significant differences were observed in the mean HbO concentration analysis among the three groups.

**Conclusion:**

This study demonstrated that auditory stimuli, especially self-referential and emotionally salient sounds, elicit distinct cortical responses in patients with different levels of consciousness.

## 1 Introduction

Disorders of consciousness (DoC) are complex clinical symptoms that are commonly observed in diseases such as brain injury, stroke, and drug poisoning (1). Prolonged disorders of consciousness (pDoC) are defined as impaired consciousness for more than 28 days (2). pDoC include coma, unresponsive wakefulness syndrome (UWS)/vegetative state (VS), and minimally conscious state (MCS) (3). The current commonly used pDoC assessment methods include behavioral scales, neuroelectrophysiological techniques, and neuroimaging techniques (4). Among them, the Coma Recovery Scale-Revised (CRS-R) is considered the “gold standard” for assessing the severity of pDoC. However, the clinician relies on the behavioral evidence exhibited by pDoC patients and the environment to determine the level of consciousness, which leads to subjective judgment by the rater. Studies have shown that the CRS-R score can reduce the misdiagnosis rate by 30–45%, and the examination of increased brain activity can further reduce the misdiagnosis rate by 30% (5). Therefore, developing a more objective and quantitative assessment of the consciousness status of pDoC patients and reducing the influence of subjective factors and the interference of external environmental factors has attracted extensive attention from scholars at home and abroad. Auditory stimulation has an important role in the assessment and diagnosis of patients with DoC (6). As a passive stimulation task, auditory stimulation does not require subjects to understand complex verbal instructions to actively cooperate and elicit a strong activation response from the cerebral cortex, has a strong arousal and emotional arousal effect, can change the perception of the environment in patients with cognitive disorders, promotes rehabilitation, and can be used as a diagnostic tool for assessing the level of consciousness (7–9). Physicians are able to use sound to assess how well a patient responds to external stimuli, for example, whether the patient is able to respond to a call sound or understand or memorize instructions or information (10). These responses indicate the patient’s level of consciousness and his or her potential for recovery (11). Specific auditory stimuli, such as the voices of family members or other familiar people, have been shown to have a positive effect on the patient’s arousal and recovery. Hearing the voices of loved ones may evoke emotional resonance and stimulate neural responses in critically ill patients and contribute to their awakening. In some clinical practices, cognitive and emotional recovery can be stimulated by repeatedly playing the voices of patients’ family members (12).

Emotions not only are an important component of consciousness but also play a key role in shaping and regulating conscious states (13). Emotions occupy a central position in consciousness, and emotions and consciousness are closely related and mutually influential. Existing research suggests that emotional auditory stimuli, including human speech, intonation, music, nature sounds, and background sounds, can elicit or stimulate specific emotional responses (14, 15). Positive emotional stimuli, such as pleasant music and the voices of loved ones, can stimulate positive emotions such as pleasure, peace of mind, and excitement, which in turn increase a person’s sense of well-being and psychological comfort (16). Negative emotional stimuli, such as angry tones, sad music, and scary sounds, may elicit negative emotional responses such as anxiety, fear, sadness, or irritability, which may lead to low mood, increased stress, or emotional instability (17, 18). Studies have shown that negative emotions are usually more likely to elicit a response than positive emotions, especially at the physiological and psychological levels (19–21). However, most of the recent research on emotional auditory stimulation has been conducted on healthy people, and less research has been conducted on the emotional awareness of patients with pDoC.

In our experiments, we used three emotional auditory stimuli: subject’s own name (SON) (22), human screams (23), and infant crying (24). Lu’s et al. (25) study have shown that name stimulation can affect prefrontal cerebral hemodynamic changes in patients with pDoC, but there is no mention of the location of the specific brain region affected. We chose fNIRS to monitor changes in cerebral blood flow in patients with pDoC. This optical technique measures changes in oxyhemoglobin (HbO) and deoxyhemoglobin (HbR) concentrations in cerebral blood flow and thus indirectly measures neural activity (26). With its good temporal [compared to functional magnetic resonance imaging (fMRI)] and spatial [compared to electroencephalography (EEG)] resolution and its non-invasive nature, fNIRS as a functional brain imaging technique is suited for monitoring cerebral oxygenation during auditory tasks (27). The prefrontal cortex plays a central role in conscious perception and is a key node within the neural network underlying awareness (28). Moreover, compared with other cortical regions, the prefrontal area is more accessible for optical measurement, being less affected by hair coverage and head/neck positioning. Given these anatomical and practical advantages, our study focused on assessing hemodynamic responses in the prefrontal cortex. fNIRS was used to measure cerebral hemodynamic changes in different brain regions of the prefrontal cortex while participants were listening to a stimulus. The aim of this study was to detect cerebral hemodynamic responses to emotional auditory stimuli in patients with DoC using fNIRS, thus providing new possibilities and research directions for assessing the level of consciousness and developing rehabilitation strategies for patients with pDoC.

## 2 Materials and methods

### 2.1 Participants

In this study, 40 patients with pDoC were recruited from Binzhou Medical University Hospital from January 2024 to July 2024. Among them, 21 patients with pDoC met the diagnostic criteria of UWS, 19 patients with pDoC were diagnosed with MCS, and all subjects met the following study enrollment criteria: (1) 18–80 years of age; (2) a state of unconsciousness resulting from a severe craniocerebral injury of more than 28 days’ duration; the included etiologies were limited to stroke and hypoxic-ischemic encephalopathy; (3) normal sleep-wake cycle; (4) diagnosed with VS/UWS or MCS according to the CRS-R scale (29); (5) patients were not hearing impaired prior to the illness, allowing for participation in auditory stimulation tasks (30); (6) no hydrocephalus or severe cerebral atrophy, and no major organ failure; (7) the patient’s guardian voluntarily participates in the study and signs an informed consent form.

Exclusion criteria for this study: (1) patients with auditory impairment; (2) patients with active cerebral hemorrhage or uncontrolled intracranial hypertension; (3) patients with a previous history of epilepsy; (4) patients with cranial defects or those who are otherwise unable to wear the fNIRS cap.

For subjects who met the inclusion criteria and volunteered to participate in this study, the initial information such as basic information was collected by 2 doctors with extensive clinical experience, and the CRS-R scale assessment was completed for pDOC patients according to a rigorous behavioral testing methodology. The clinical characteristics of the patients are shown in Table 1.

**Table 1:** Clinical features of patients with pDoC.

Clinical Features	VS/UWS	MCS	*p*-value
Age (years)	58.5 ± 5.5	56.2 ± 7.4	0.151
Sex (male/female)	16/4	13/6	0.408
Months since injury	2.9 ± 1.1	2.6 ± 0.9	0.926
CRS-R total score	3.7 ± 1.1	13.4 ± 2.4	0.010^*^

^*^p < 0.05.

### 2.2 Study design

**Figure 1:**
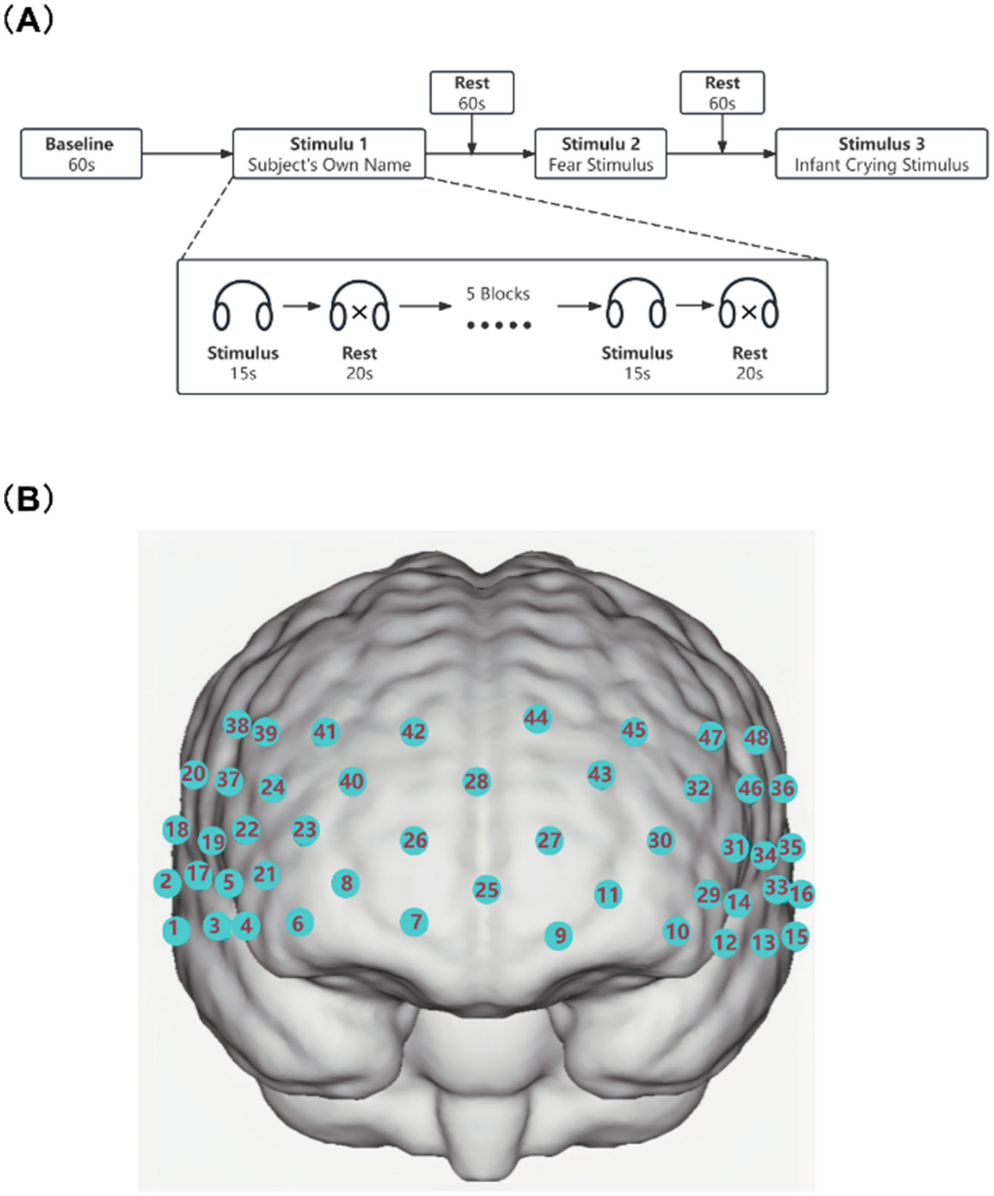
**(A)** Experimental protocols. **(B)** Schematic representation of channel positioning on the scalp. Images were created using NirSpark software.

Subjects were exposed to three different auditory stimuli: a SON stimulus, a fear stimulus (human scream), and a baby crying stimulus. For the auditory stimuli, the voice words were synthesized using standard Mandarin speech at medium speed and 70 decibels (dB) through professional dubbing software, all the auditory stimulus were adjusted to mid-frequency sounds at the same volume (25, 30). The sounds are stored in WAV format and the auditory stimuli will be presented by the Nirscan software and played through headphones. The specific experiment is the block task paradigm. The experimental paradigm consists of an initial baseline and three stimulus tasks, each with five block tasks (25). Each block consisted of a task cycle (15 s) and a rest cycle (20 s) (31). A rest period (20 s) was also included between neighboring different auditory stimulus tasks. The experimental protocol for this study is shown in Figure 1A.

### 2.3 Data acquisition and analysis

#### 2.3.1 Data acquisition

In this experiment, a NirScan-6000A (Danyang Huichuang Medical Equipment Co., Ltd., China) was used to continuously measure and record changes in the concentrations of HbO and HbR in the brain during the tasks. The system included a near-infrared light source [light emitting diodes (LEDs)] and avalanche photodiodes (APDs) as detectors, with wavelengths of 730 nm, 808 nm and 850 nm and a sampling rate of 11 Hz. Fifteen light sources and 16 detectors were combined to form 48 effective channels, and the average distance between the source and the detector was 3 cm (range: 2.7–3.3 cm). According to the international 10/20 system for positioning, we used a 3D digitizing system to determine Montreal Neurological Institute (MNI) coordinates for each participant (Figure 1B).

#### 2.3.2 Data preprocessing

fNIRS signal preprocessing: fNIRS signal preprocessing was conducted with NirSpark software (Danyang Huichuang Medical Equipment Co., Ltd., China). HbO was used as the main parameter reflecting hemodynamic changes during the task. The raw data were first processed with NirSpark software, which includes artifact processing, filtering, segmentation, and baseline comparison, and the raw near-infrared spectral intensity signals were converted to signals and then to oxygen concentration data. The data were statistically analyzed using the average value of each channel, and then the built-in preprocessing module of the software was used to preprocess the collected near-infrared spectroscopy (NIR) spectral data, with the threshold standard deviation set to 6.0 and the amplitude threshold set to 0.5. Motion artifacts were removed via threefold spline interpolation combined with the offset of the standard deviation (32, 33), and the signal interference caused by the signals from the heart rate and respiratory rate and the Mayer wave were removed via bandpass filtering from 0.01–0.20 Hz (34), and the differential path length factor was set to 6.0 (35). Signal interference caused by differential filtering was removed based on the difference between the signal and the respiratory frequency. The modified Beer–Lambert law was subsequently used to calculate the relative changes in the concentrations of HbO and HbR. The random noise between tasks was eliminated by superimposing the block paradigm and averaging to obtain the hemodynamic response function (HRF). The initial time of the HRF was set to −2 s to 0 s, and the end time was set to 15 s. The oxygenated HRF was averaged for each channel across the four blocks. The general linear model (GLM) was used to establish an ideal HRF for each task for each subject, and the degree of matching between the experimental and ideal HRF values was calculated. For feature extraction, we selected two types of hemodynamic indicators: the block-averaged mean and the slope of the response. The mean value reflects the magnitude of cortical activation, representing the overall intensity of the brain’s response to auditory stimuli. In contrast, the slope captures the rate of change in the hemodynamic signal, providing information about the speed and dynamics of the neural response (36, 37). Then, the baseline values were subtracted from the data between −2 s before stimulation and 35 s after stimulation, and the brain functional response curves of the HbO signals in the task interval were obtained. In the domain of fNIRS, mean and slope values are frequently employed as biomarkers during fNIRS feature extraction (38). Consequently, the present study extracted the mean and slope value of the hemodynamic responses during the stimulation period (0–15 s) to investigate the differences between the UWS, MCS and HC group.

### 2.4 Statistical analysis

Single-channel data processing and analysis were performed using SPSS 25.0 statistical software and statistical graphs were presented using GraphPad Prism 10.0 software. Data were categorized into UWS, MCS, and HC according to the level of consciousness. The Shapiro–Wilk test revealed non-normal distribution of the data (*p* < 0.05), necessitating non-parametric statistical approaches. Between-group comparisons were conducted using the Kruskal-Wallis test, with Dunn’s *post hoc* test (Bonferroni-corrected) for pairwise comparisons when significant main effects were detected. Statistical significance was set at *p* < 0.05 (two-tailed).

## 3 Results

### 3.1 SON stimulus—mean value analysis

**Figure 2:**
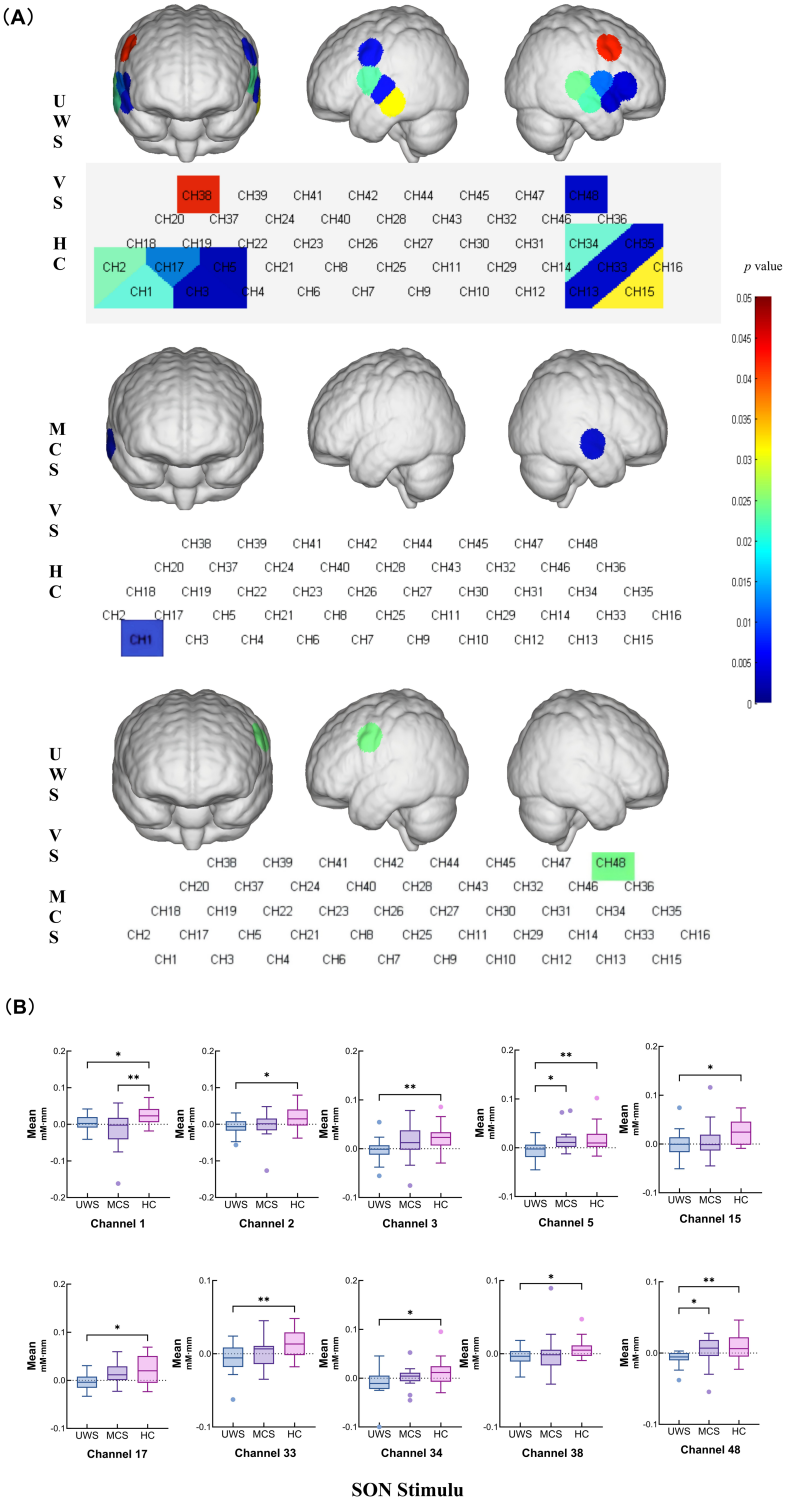
Intergroup comparisons of the mean values of the UWS, MCS and HC groups. **(A)** Differences in channel waveforms between pDoC and HC subjects with the SON stimulus. **(B)** Box plots of significant differences in the mean hemodynamic changes among the UWS, MCS, and HC groups during the SON task (^*^p < 0.05 and ^**^p < 0.01).

During the SON task, the mean hemodynamic changes at channel 1 (*p* = 0.0225), channel 2 (*p* = 0.0242), channel 3 (*p* = 0.0041), channel 5 (*p* = 0.0026), channel 15 (*p* = 0.0310), channel 17 (*p* = 0.0114), channel 33 (*p* = 0.0059), channel 34 (*p* = 0.0230), channel 38 (*p* = 0.0419), and channel 48 (*p* = 0.0051) were significantly lower in the UWS group than those in the HC group. At channel 1 (*p* = 0.0029), the mean hemodynamic changes were significantly lower in the MCS group than in the HC group. Channel 48 (*p* = 0.0243) showed significantly greater mean hemodynamic changes in the MCS group than in the UWS group (Figures 2A,B and Table 2).

**Table 2:** Information on statistically significant channels.

Channel	S-D	MNI coordinate system			Brodmann areas	Left/Right (L/R)
		X	Y	Z		
CH 1	S1-D1	70.26	−11.02	−10.66	MTG	R
CH 2	S1-D6	71.33	−24.44	1.531	MTG	R
CH 3	S2-D1	59.98	12.26	−10.26	TPA	R
CH 5	S2-D7	59.04	26.43	0.6173	PTG	R
CH 15	S6-D5	−72.35	−22.24	−12.9	MTG	L
CH 17	S7-D1	64.4	1.181	1.752	RSA	R
CH 28	S9-D14	1.327	64.37	24.94	FPA	
CH 32	S10-D15	−49.52	43.11	23.11	TPA	L
CH 33	S11-D5	−69.63	−11.55	−1.151	MTG	L
CH 34	S11-D10	−65.45	2.72	8.454	RSA	L
CH 38	S12-D12	57.21	9.608	39.39	SMA	R
CH 43	S14-D9	−26.85	62.43	26.24	DLPFC	L
CH 44	S14-D14	−12.31	58.91	40.02	DLPFC	L
CH 48	S15-D16	−63.43	2.604	35.99	SMA	L

MTG, middle temporal gyrus; TPA, temporopolar area; PTG, pars triangularis Broca’s area; RSA, retrosubicular area; FRA, frontopolar area; SMA, premotor and supplementary motor area; DLPFC, dorsolateral prefrontal cortex.

### 3.2 Fear stimulus—mean value analysis

**Figure 3:**
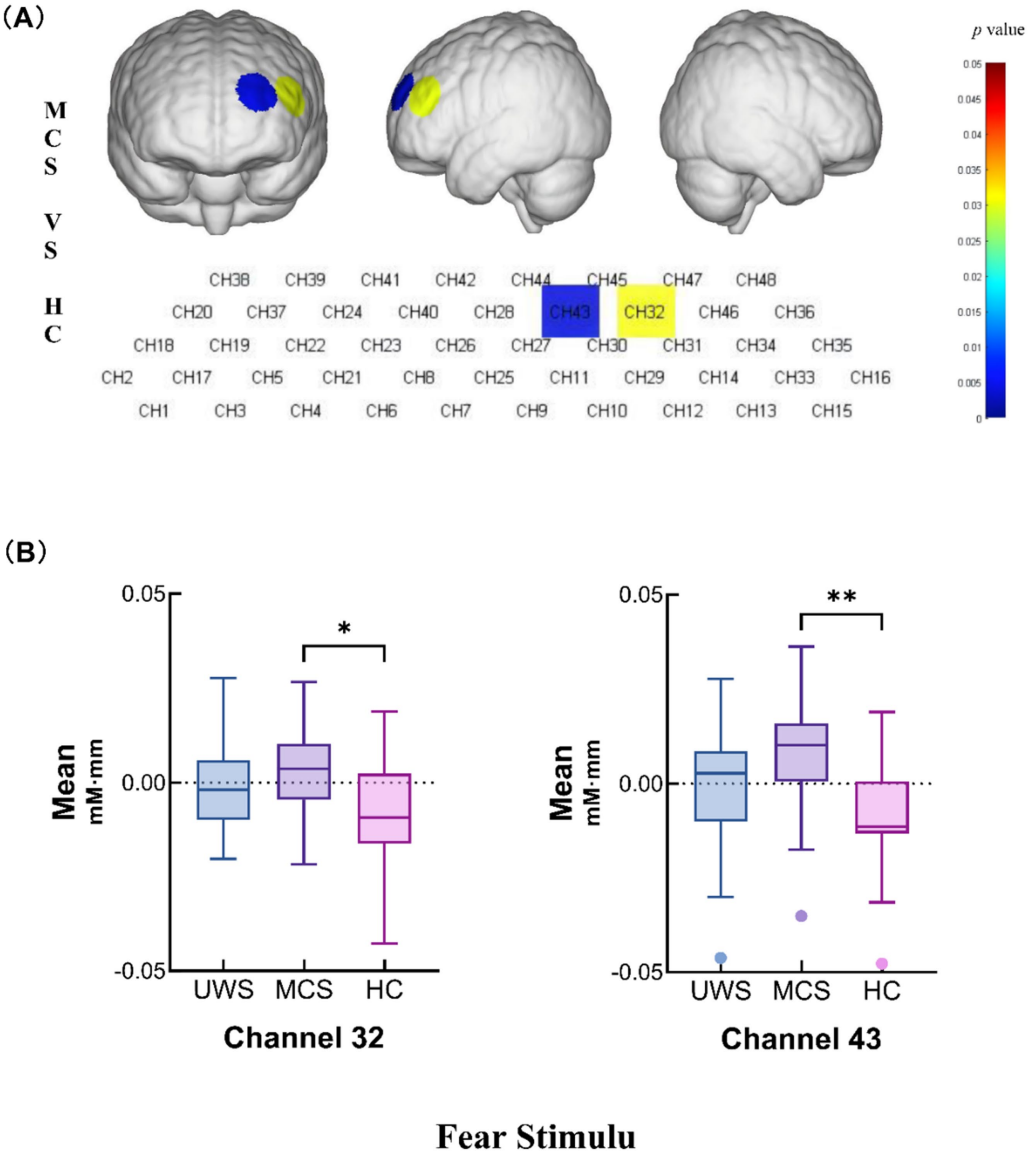
Intergroup comparisons of the mean values of the UWS, MCS and HC groups. **(A)** Differences in channel waveforms between pDoC and HC subjects with the fear stimulus. **(B)** Box plots of significant differences in the mean hemodynamic changes among the UWS, MCS, and HC groups during the fear stimulation task (*p <0.05 and **p < 0.01).

In the fear stimulation task, the MCS group demonstrated significantly greater mean hemodynamic changes at channel 32 (*p* = 0.0297) and channel 43 (*p* = 0.0040) compared with those in the HC group. These findings suggest that individuals in the MCS group exhibit a more pronounced hemodynamic response when subjected to fear stimuli (Figures 3A,B and Table 2).

### 3.3 Fear stimulus—slope analysis

**Figure 4:**
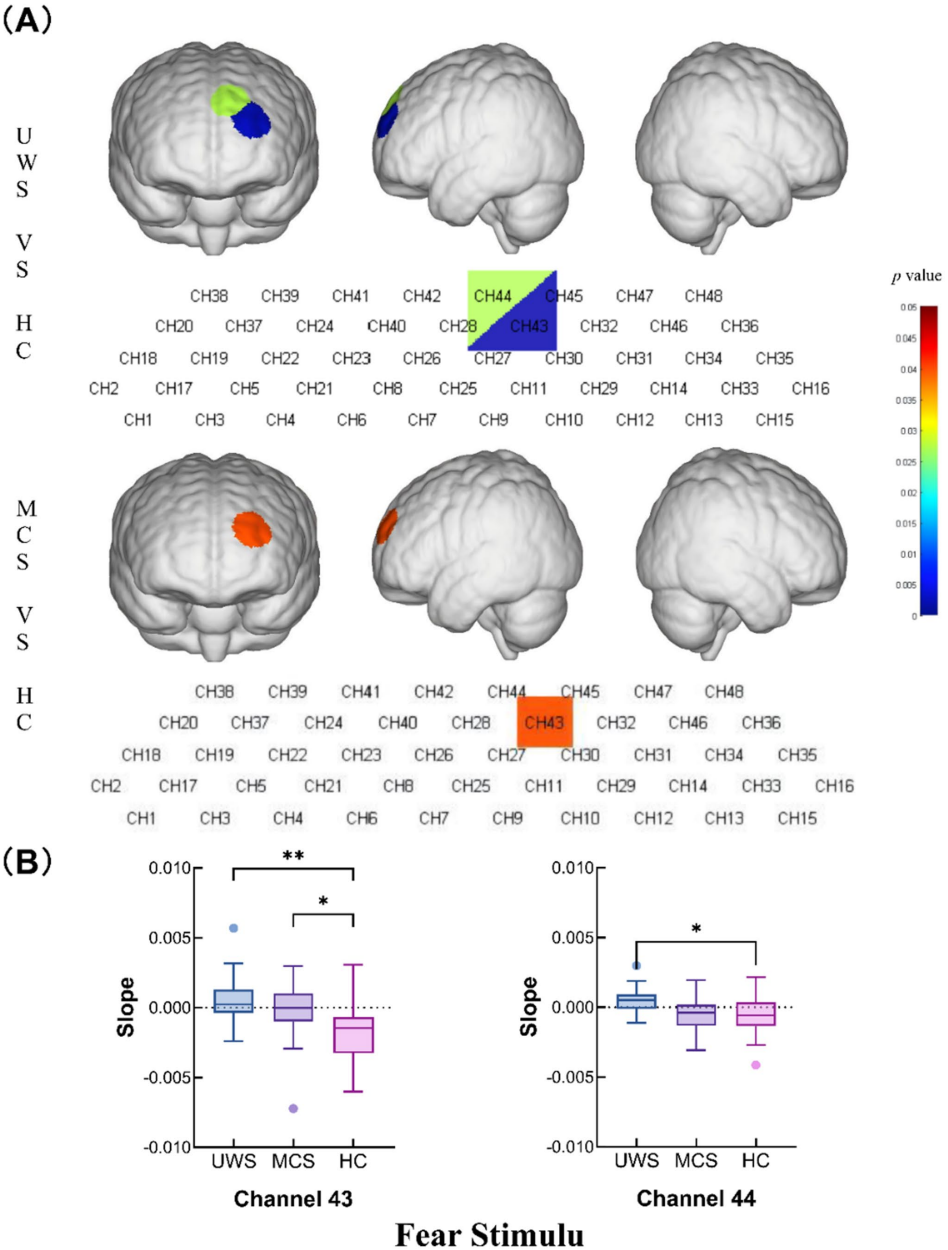
Intergroup comparison of the slopes of the UWS, MCS and HC groups. **(A)** Differences in channel waveforms between pDoC and HC subjects with the fear stimulus. **(B)** Box plots of significant differences in the changes in the slope of the hemodynamic curve among the UWS, MCS, and HC groups during the fear stimulation task (*p < 0.05 and **p < 0.01).

Slope analysis of the curve for the fear stimulation task revealed that channels 43 (*p* = 0.0012) and 44 (*p* = 0.0272) exhibited a significantly greater change in the hemodynamic curve slope in the UWS group than in the HC group. Additionally, the MCS group demonstrated a significantly greater change in slope at channel 43 (*p* = 0.0399) than the HC group did, indicating a heightened sensitivity to fear-related stimuli in the MCS group (Figures 4A,B and Table 2).

### 3.4 Infant crying stimulus—slope analysis

**Figure 5:**
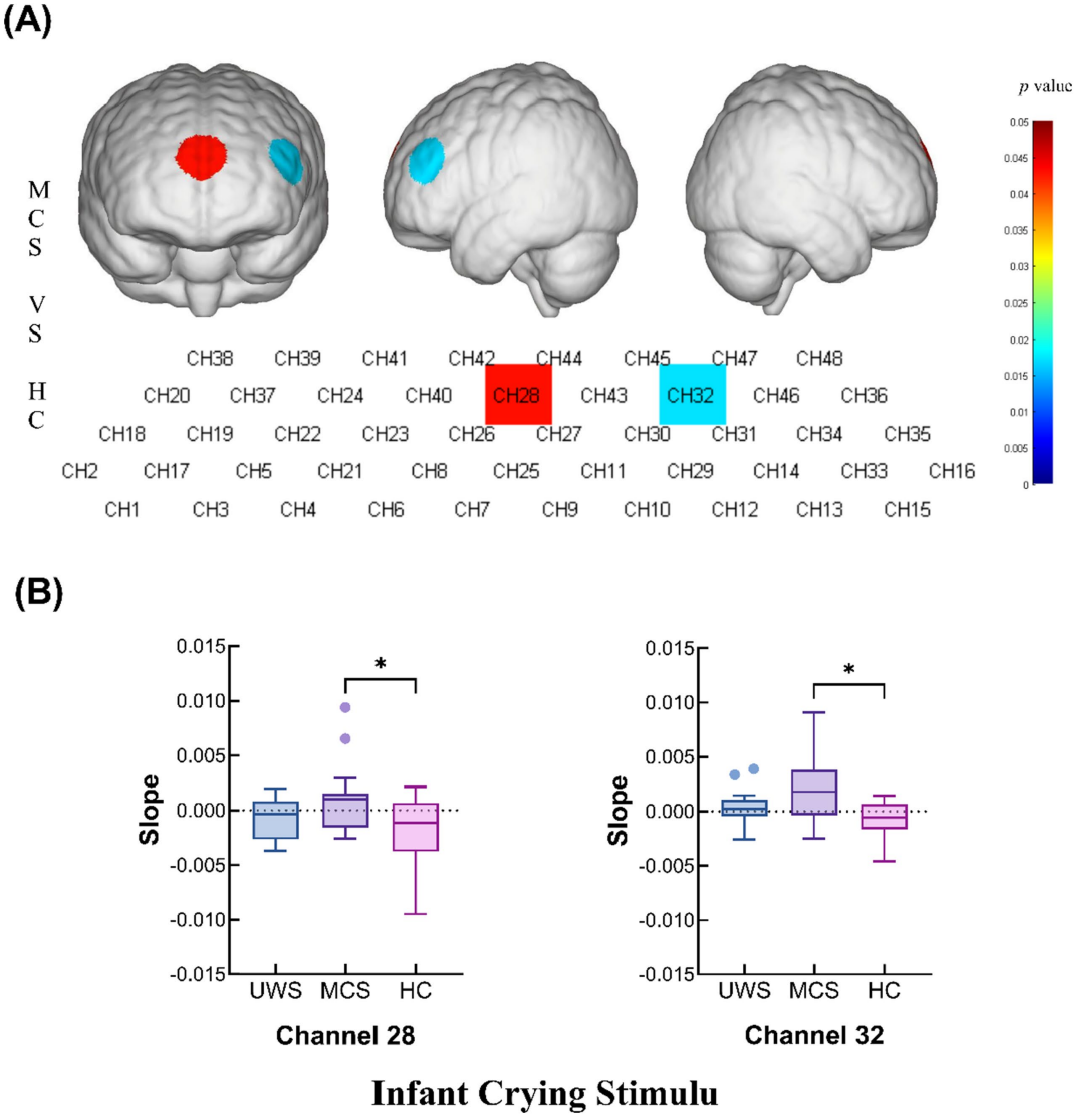
Intergroup comparison of the slopes of the UWS, MCS and HC groups. **(A)** Differences in channel waveforms between pDoC and HC subjects with the infant crying stimulus. **(B)** Box plots of significant differences in the changes in the slope of the hemodynamic curve among the UWS, MCS, and HC groups during the infant crying task (*p < 0.05 and **p < 0.01).

During the infant crying stimulation task, the MCS group presented significantly greater changes in the slope of the hemodynamic curve at channels 28 (*p* = 0.0429) and 32 (*p* = 0.0172) compared with those in the HC group. These findings suggest that the MCS group exhibits a more robust hemodynamic response to infant crying stimuli (Figures 5A,B and Table 2).

## 4 Discussion

In this study, fNIRS was used to evaluate the effect of emotional auditory stimulation on cerebral blood flow in patients with pDoC. The complex etiologic and neurobiological mechanisms underlying pDoC, as well as individual differences and fluctuations in arousal between patients, may contribute to differences in subject-specific hemodynamic responses (39). In a comparative study of 60 patients with pDoC and HCs, we found that different types of auditory stimuli had significantly different effects on cerebral blood flow in patients with UWS and MCS. These findings provide new insights into the residual consciousness and emotional processing of patients with pDoC.

### 4.1 SON stimulus in auditory processing

The use of the SON as an auditory stimulus has been shown to elicit significant neural responses in healthy individuals and, to a lesser extent, in pDoC patients (10, 40, 41). The results revealed that the mean blood oxygen concentration during the SON stimulus in several brain regions, particularly the bilateral MTG (channels 1, 2, 15, 17 and 33), RSA (channels 17 and 34), SMA (channels 37 and 48) and right PTG (channel 5), and TPA (channel 3), which are associated with language processing, was significantly greater in the HC group than in the UWS group. Our findings are consistent with those of previous studies that have reported that self-referential stimuli elicit strong activation of the cerebral cortex, especially in the MTG and Broca’s area, in healthy individuals (42, 43). This response is often considered an indication that consciousness is preserved, even in people with pDoC, because the stimulus touches on emotions and personal meaning (44). Our findings show that activation was significantly greater in the HC group than in the UWS group at multiple channels, especially in the temporal region. This enhanced response may be due to the intrinsic salience and emotional importance of the SON, which mobilizes both cognitive and emotional processing networks (45).

Interestingly, MCS patients demonstrated a greater response at the left SMA than UWS patients did, which corresponds to the premotor and supplementary motor cortices, suggesting that even in states of minimal consciousness, self-referential stimuli can evoke detectable responses. The significant differences at channel 48 may be related to movement preparation and exercise programs. Hearing one’s name may trigger potential movement readiness (e.g., turning one’s head in response), which was particularly evident in the HC group (45, 46). In contrast, activation in this region in the MCS group was lower than that in the HC group but still significantly greater than that in the UWS group, suggesting that MCS patients may still have some capacity for motor readiness after hearing their name, which further supports the residual level of consciousness in MCS patients. Names appear to be very effective attention-grabbing stimuli that break through barriers and engage consciousness first. These findings are consistent with those of previous studies in which MCS patients exhibited preserved affective processing. These results support the theory that auditory stimuli, particularly self-referential stimuli, could serve as valuable diagnostic tools for assessing residual awareness in pDoC patients (44, 47).

### 4.2 Fear and infants crying stimuli: the role of emotional salience

The emotional salience of fear-inducing stimuli, such as a woman’s scream, elicited significant activation in both the UWS and MCS groups, particularly in the left DLPFC (channels 43 and 44). Fear sounds are nonverbal human sounds that can elicit the emotion of fear in normal, healthy people by triggering the amygdala, and previous research has shown that negative emotional sounds, such as screaming or crying, engage both the auditory cortex and emotional processing areas such as the amygdala (48). The DLPFC has an extensive functional network that connects this region to several subcortical regions of the brain, such as the amygdala and midbrain regions (49). The emotional function of the amygdala has been demonstrated in numerous studies (50, 51). The heightened response in MCS patients relative to HCs at some channels suggests that negative emotional stimuli may be more effective in capturing attention and processing than neutral or less emotionally charged stimuli.

Moreover, UWS patients displayed greater activation in response to fear stimuli than HCs did in the left DLPFC (channels 43 and 44). This finding is consistent with the idea that certain emotional stimuli might bypass higher-order cognitive networks, engaging more primitive affective circuits that remain functional even in severe pDoC.

Our findings show that compared with HCs, MCS patients elicited greater changes in slope values at channels 28 and 32, which represent frontal regions associated with emotion regulation and decision-making, suggesting that infant crying sounds can have a strong emotional and attentional impact on adults. The higher slope values in the MCS group than in the HC group could reflect heightened sensitivity to emotionally charged, biologically relevant stimuli, a pattern that has been observed in other studies exploring emotional and auditory processing (52, 53).

These findings suggests that the absence of behavioral responsiveness does not necessarily equate to diminished cortical reactivity, especially when stimuli are emotionally salient and biologically relevant. From a neurobiological perspective, emotionally charged stimuli, such as fear-related sounds, are known to activate subcortical-limbic circuits (e.g., amygdala, thalamus), which then project to prefrontal regions (notably the DLPFC) involved in emotional appraisal and cognitive modulation (54). In MCS patients, partial integrity of this amygdala-DLPFC pathway may persist despite global network disruption, allowing for residual, and potentially exaggerated, responses to salient inputs. This is supported by prior research showing that subcortical and emotional systems are relatively more preserved than language or motor networks in pDoC (55). Moreover, the enhanced activation in MCS patients could reflect neuroplasticity-driven compensatory reorganization. After focal or diffuse brain injury, surviving regions may take on greater functional load, leading to hyperactivation when stimulated.

An alternative explanation involves disinhibition: damage to inhibitory prefrontal areas (e.g., medial PFC or anterior cingulate cortex) may reduce top-down control, allowing emotionally salient signals to evoke amplified bottom-up activity (56). This may manifest as stronger hemodynamic signals in regions like the DLPFC, even if the patient lacks overt behavioral output.

Taken together, these findings suggest that emotional auditory stimulus can tap into covert affective processing circuits in MCS and even UWS patients. Rather than being noise or artifact, such hyper-responsivity may serve as a neurophysiological marker of residual consciousness—offering both diagnostic and therapeutic utility. Future work integrating fNIRS with fMRI and EEG connectivity analyses could further clarify the reorganization of emotion-related brain networks in pDoC.

### 4.3 Cortical asymmetry and emotional processing

The asymmetry in cortical activation observed in this study, particularly the dominance of activation in certain channels in response to emotional stimuli, mirrors the findings of previous studies investigating the lateralization of emotional processing (57, 58). The valence hypothesis posits that positive and negative emotions differentially engage the left and right prefrontal cortex, respectively. In our study, fear stimuli predominantly activated left-lateralized regions, which is consistent with the idea that the left hemisphere is more involved in processing negative emotional cues (23). This lateralization may reflect the functional reorganization or compensatory activation patterns in the injured brain. In MCS patients, enhanced left prefrontal responses may indicate residual capacity for higher-order emotional and cognitive processing that remains latent in UWS patients. It is also plausible that asymmetric structural damage in the right hemisphere could lead to a relative functional dominance of the left hemisphere in response to external stimuli.

### 4.4 Implications for diagnostic and therapeutic approaches

These findings offer promising insights into the potential of using fNIRS as a tool for assessing residual cognitive and affective processing in patients with pDoC. The differential activation patterns among the UWS, MCS, and HC groups in response to self-referential and emotionally salient auditory stimuli could aid in the development of more nuanced diagnostic criteria, helping clinicians better distinguish between levels of consciousness.

Additionally, the ability of emotionally charged stimuli to evoke stronger cortical responses in MCS patients suggests that these stimuli could be used in therapeutic contexts to engage patients more effectively, potentially aiding in the recovery of consciousness. This study further demonstrates the feasibility and usability of the fNIRS-based emotional auditory stimulation paradigm for detecting residual consciousness in pDoC patients, providing a basis for the assessment and treatment of emotional sounds in patients with pDoC.

## 5 Limitations and future directions

The current study has several important limitations. (1) It is important to note that this study focused exclusively on the prefrontal region and part of the temporal lobe. In addition, due to the relatively small sample size, we did not perform stratified analyses based on etiology, which may limit the interpretation of differential responses associated with specific types of brain injury. Future studies with larger cohorts should consider full-head fNIRS coverage and stratified analysis by etiology to more comprehensively assess the cerebral responses of pDoC patients to external stimuli. (2) The etiology of pDoC is multifaceted, and in subsequent exploratory studies, the differences in etiology and damaged brain regions among subjects should be taken into account. Furthermore, the relationship between cerebral hemodynamic changes and damaged brain regions should be investigated in conjunction with fMRI. (3) Quantitative correlation analysis between the feature value and CRS-R scores was lacking in this study. This limits our ability to directly assess the added diagnostic value or predictive accuracy of fNIRS measures in relation to established behavioral assessments. In future studies, we aim to perform correlational and regression analyses between fNIRS features (e.g., mean, slope) and CRS-R scores. (4) While this study employed traditional statistical methods to compare hemodynamic responses across groups, we acknowledge that more advanced data analysis techniques, such as machine learning (ML) algorithms, may offer additional insights. Given the exploratory nature of our study and the relatively small sample size, we prioritized interpretable and well-established metrics (i.e., mean and slope of HbO signals) to ensure interpretability and clinical relevance. However, we recognize that ML methods—such as support vector machines, random forests, or deep learning—could enhance the classification and predictive power of fNIRS data. Future studies with larger, multi-center cohorts and richer datasets may benefit from the integration of ML-based approaches to improve diagnostic accuracy and uncover latent biomarkers of consciousness. This represents a promising direction for the continued development of fNIRS-based assessment tools in clinical settings.

## 6 Conclusion

This study provides preliminary evidence that auditory stimuli, particularly self-referential and emotionally salient sounds, may elicit differential cortical responses in patients with varying levels of consciousness. The observed patterns tentatively suggest that emotionally salient sounds could potentially engage neural circuits associated with emotional processing, even in patients with disorders of consciousness. While these findings are consistent with the hypothesis that emotional auditory stimuli might help assess residual cognitive-emotional function in pDoC patients, further validation in larger cohorts is needed to confirm these observations and establish their clinical relevance.

## Data Availability

The raw data supporting the conclusions of this article will be made available by the authors, without undue reservation.
